# 固相萃取净化-超高效液相色谱-串联质谱法同时测定预制菜食品中15种双酚类化合物

**DOI:** 10.3724/SP.J.1123.2024.11014

**Published:** 2025-10-08

**Authors:** Ziyue ZHAN, Qi ZHANG, Shuangshuang TIAN, Ziwei ZHAO, Yanyu DAI, Bolin LIU

**Affiliations:** 安徽省疾病预防控制中心，安徽 合肥 230601; Anhui Provincial Center for Disease Control and Prevention，Hefei 230601，China

**Keywords:** 双酚类化合物, 超高效液相色谱-串联质谱法, 预制菜食品, 固相萃取, bisphenols （BPs）, ultra performance liquid chromatography-tandem mass spectrometry （UPLC-MS/MS）, prepared dishes, solid phase extraction

## Abstract

双酚类化合物（BPs）被广泛用于制备环氧树脂和聚碳酸酯塑料，常见于食品包装和饮料容器中，其从食品包装材料向食品的迁移风险已成为重要的研究课题。鉴于此，本文基于超高效液相色谱-串联质谱（UPLC-MS/MS）建立了一种高效、简洁、准确的可同时测定预制菜食品中15种BPs含量水平的测定方法。粉碎混合均匀的预制菜样品经2.0 mL超纯水分散后再加入8.0 mL乙腈进行涡旋超声振荡提取，取离心后的上清液过Captiva EMR-Lipid净化柱。以甲醇-0.01%（v/v）氨水溶液为流动相，流速为0.2 mL/min，采用Waters ACQUITY HSS T_3_色谱柱（100 mm×2.1 mm，1.8 μm）以及Waters ACQUITY BEH C_18_捕集柱（50 mm×2.1 mm，1.7 μm）对目标物进行分离。在电喷雾电离负离子模式（ESI^-^）和多反应监测（MRM）模式下采集质谱数据，同位素内标法定量。在优化的实验条件下，15种BPs在各自的线性范围内具有良好的线性关系，相关系数（*R* ^2^）均大于0.999 0，方法检出限为0.01～0.45 μg/kg，定量限为0.03～1.50 μg/kg。采用低、中、高3个加标水平考察方法的准确性与精密度，15种BPs的加标回收率为70.9%～105.8%，相对标准偏差（RSD）为0.6%～9.1%（*n*=6）。将建立的方法用于分析测定30份预制菜样品，结果表明：共有6种BPs被检出，分别为双酚A（BPA）、双酚B（BPB）、双酚C（BPC）、双酚G（BPG）、双酚S（BPS）和双酚AF（BPAF），其中BPA的检出率最高，为16.7%，其次BPC、BPG、BPB、BPAF和BPS的检出率分别为10.0%、10.0%、6.67%、6.67%和3.33%。该方法前处理简单，精密度好，灵敏度高，可对预制菜食品中15种BPs进行准确定性定量分析。

## Abstract (en)

Bisphenols （BPs）， which include bisphenol A and its analogs （such as bisphenol S， bisphenol AF， and bisphenol B）， are chemical substances that are synthesized artificially. BPs are used in epoxy resins and polycarbonate plastics and are widely found in food packaging and beverage containers. BPs are endocrine disruptors； hence， they can disrupt the natural hormonal activities of the human body， impact the neurological development of children， and affect intestinal microbial communities in the body， leading to obesity. Consequently， BPs pose threats to human health. The prepared dishes are usually using plastic packaging （containers， films， tubes， etc.）； consequently， BPs migrate from the packaging material to the food in the container is hot topic of wide concern. To address this issue， an efficient， simple and accurate method for the simultaneous determination of the 15 BPs content levels in prepared dishes using ultra performance liquid chromatography-tandem mass spectrometry （UPLC-MS/MS） was developed. The effects of the extraction solvent and its proportion， the amount of weighed matrix， and type of purifying agent on the responses and recoveries of the 15 BPs were investigated under the optimized MS conditions with the aim of determining the optimal sample-preparation conditions. The prepared dishes samples were crushed and evenly mixed， after which an internal standard solution containing a mixture of bisphenols was added and dispersed using 2.0 mL of ultrapure water. Acetonitrile （8.0 mL） was subsequently added， and the sample was extracted using vortex ultrasonication， centrifuged at 10 000 r/min for 10 min， and a 3.0-mL aliquot of the centrifuged supernatant was cleaned using a Captiva EMR-Lipid clean-up column， after which the purified liquid was subjected to UPLC-MS/MS. Analyses of response values， separation effects， and chromatographic peak shapes revealed that the Waters ACQUITY HSS T_3_ column （100 mm×2.1 mm， 1.8 μm） optimally separated the 15 target BPs at a column temperature of 40 ℃， a flow rate of 0.2 mL/min， and an injection volume of 2.0 μL. A Waters ACQUITY BEH C_18_ column （50 mm×2.1 mm， 1.7 μm） was used to trap and eliminate interference from exogenous BPs in the piping components. Gradient elution was performed using methanol and 0.01% （v/v） aqueous ammonia as mobile phases. Data were collected in electrospray negative-ion （ESI^-^） and multiple reaction monitoring （MRM） modes， and quantified using the isotope internal standard method. The 15 BPs exhibited good linear relationships within their respective linear ranges under the optimized experimental conditions， with correlation coefficients （*R* ^2^） greater than 0.999 0. The limits of detection （LODs） and limits of quantification （LOQs） were in the range of 0.01-0.45 μg/kg and 0.03-1.50 μg/kg， respectively. The recoveries and relative standard deviations （RSDs） of the 15 BPs in matrix sample of prepared dishes at low， medium， and high spiked levels were 70.9%‒105.8% and 0.6%‒9.1% （*n*=6）， respectively. The established method was used for the analytical determination of the 15 BPs in 30 prepared dishes samples， bisphenol A， bisphenol B， bisphenol C， bisphenol G， bisphenol S and bisphenol AF were detected in 11 samples， with bisphenol A exhibiting the highest detection rate of 16.7%， followed by bisphenol C， bisphenol G， bisphenol B， bisphenol AF， and bisphenol S， with values of 10.0%， 10.0%， 6.67%， 6.67% and 3.33%， respectively. The median contents of bisphenol G， bisphenol B， bisphenol A， bisphenol C， bisphenol S， and bisphenol AF were 4.15， 3.25， 2.68， 2.16， 1.49， and 0.47 μg/kg， respectively， with two BPs detected in 16.7% of the samples. The developed method involves simple pretreatment， is highly precise and sensitive， and is capable of accurately and qualitatively analyzing the 15 BPs in prepared dishes.

双酚类化合物（BPs）是指一类两个羟苯基由一个或多个碳原子连接起来的人工合成化合物^［1］^，是一类重要的有机化工原料，包括双酚A（BPA）、双酚B（BPB）、双酚C（BPC）、双酚S（BPS）、双酚F（BPF）、双酚E（BPE）、双酚Z（BPZ）、双酚AP（BPAP）、双酚AF（BPAF）、四溴双酚A（TBBPA）等，主要用于生产聚碳酸酯、环氧树脂、不饱和聚酯树脂等多种高分子材料^［2］^，也可用于生产增塑剂、阻燃剂、抗氧剂、热稳定剂等精细化工产品^［3］^，广泛存在于食品包装、饮料容器、玩具及个人护理产品中。BPA是用途最广泛的BPs，流行病学与实验研究表明，因BPA具有内分泌干扰物的作用，会扰乱人体的天然荷尔蒙活动，影响儿童神经发育和体内肠道微生物群落，以及导致肥胖等^［4，5］^，损害人体健康，因此，BPA产生的健康风险引起了人们的广泛关注。越来越多的国家或组织限制BPA的使用^［1，2］^，但其替代物被广泛应用，越来越多的报道表明，BPA及其替代物已在食品中检出^［6，7］^，如果蔬^［8］^、罐头食品^［9］^、谷类^［10］^、水产类^［3］^、蛋类^［11］^等。这些替代化合物具有类似BPA的化学结构和性质^［12，13］^。研究表明，这些双酚类化合物仍然存在内分泌干扰物作用^［14］^，可导致生殖功能障碍^［15，16］^、神经系统发育受制^［17］^、甲状腺功能异常^［18］^、肾功能水平低下^［19］^、肥胖^［20］^等诸多不良影响。

预制菜是以一种或多种食用农产品及其制品为原料，使用或不使用调味料等辅料，经工业化预加工制成，配以或不配以调味料包，加热或熟制后方可使用的预包装菜肴食品^［21-23］^。因此，预制菜在销售过程中要经过包装等工序，包装材料在生产的过程中产生BPs残留^［2］^，在高温、酸性或油脂等条件下，BPs更容易从包装材料中释放出来^［24］^，并迁移到食品中，尤其是定型包装食品经过二次加热后，包装材料中的BPs易通过膳食途径进入人体，人体暴露于高含量的BPs，其健康风险不容小视。

目前，针对原型食品中双酚类化合物的检测方法已有报道^［25，26］^，高效液相色谱-串联质谱法因其灵敏度高、分析时间短、试剂消耗小、受基质干扰因素小、分离度好，并兼具定性定量等特点，广泛用于食品中BPs含量的测定^［23］^。韩沐珂等^［27］^采用免疫亲和柱净化浓缩样品提取液，建立了高效液相色谱-串联质谱法测定植物源食品中的双酚类化合物。王斌等^［10］^采用正己烷脱脂，基质分散固相萃取净化样品，开发了液相色谱-串联质谱法检测烘焙食品及原料中BPs的分析方法，克服了馅料中油脂对测定的干扰。赵斌等^［8］^采用QuEChERs法处理水果和蔬菜样品，结合超高效液相色谱-串联质谱法（UPLC-MS/MS）快速测定8种双酚类物质。

Captiva EMR-Lipid柱净化法为“一步法”净化，在除复杂脂肪样品中的脂质、脂肪和其他干扰物质方面具有一定优势^［28，29］^，适合预制菜样品的前处理净化。本研究以预制菜为基质，选择BPA及替代物等15种BPs为研究目标物，乙腈作为提取液，使用Captiva EMR-Lipid净化柱净化，结合UPLC-MS/MS，同位素内标法定量。经高效除脂净化后，降低了基质效应，15种BPs具有较好的回收率，实现了预制菜食品中15种BPs的定性和定量测定。该方法前处理步骤绿色环保，简单快捷，准确度和灵敏度高，适用于预制菜食品中15种BPs的快速测定。

## 1 实验部分

### 1.1 仪器、试剂与材料

PREMIER超高效液相色谱仪、三重四极杆质谱仪（Xevo TQ-XS）（美国Waters公司）；Legend Mach 1.6R冷冻离心机（美国Thermo Fisher公司）；Multi Reax多试管高速振荡器（德国Heidolph公司）；Milli-Q超纯水系统（美国Millipore公司）；AS系列超声波清洗机（天津奥特赛恩斯仪器有限公司）。

甲醇、乙腈为色谱级，购于美国Merck公司；乙二胺-*N*-丙基硅烷化硅胶（PSA）、C_18_吸附剂（50 μm）、Captiva EMR-Lipid净化柱（3 mL/300 mg）均购自美国Agilent公司；Oasis PRiME HLB固相萃取柱（3 mL/60 mg，美国Waters公司）。双酚类化合物标准品：BPA（CAS号：80-05-7）、BPB（CAS号：77-40-7）、BPC（CAS号：79-97-0）、BPE（CAS号：2081-08-5）、BPF（CAS号：620-92-8）、双酚G（BPG，CAS号：127-54-8）、双酚M（BPM，CAS号：13595-25-0）、双酚P（BPP，CAS号：2167-51-3）、BPS（CAS号：80-09-1）、BPZ（CAS号：843-55-0）、BPAP（CAS号：1571-75-1）、BPAF（CAS号：1478-61-1）、双酚BP（BPBP，CAS号：1844-01-5）、双酚FL（BPFL，CAS号：3236-71-3）、TBBPA（CAS号：79-94-7）纯度均大于98%；双酚类化合物内标：^13^C_12_-BPA、D_8_-BPB、D_12_-BPE、D_10_-BPF、D_8_-BPS、D_6_-BPZ、D_5_-BPAP、^13^C_12_-BPAF、D_6_-TBBPA纯度大于 99.0%，以上均购自英国LGC公司。

### 1.2 溶液配制

分别准确称取15种BPs及9种同位素内标（精确至0.1 mg），用甲醇稀释配制，得到各目标物储备液的质量浓度为1.0 mg/mL，同位素内标储备液的质量浓度为0.5 mg/mL，存放在‒18 ℃冰箱中备用。

将上述各标准储备液用80%（v/v）乙腈水溶液配制成混合标准使用液和混合同位素内标使用液后，用80%（v/v）乙腈水溶液逐级稀释成系列标准溶液，其中BPM、BPP、BPS、BPAF的质量浓度为0.02~10.0 ng/mL，BPZ、BPBP、BPFL、BPAP的质量浓度为0.04~20.0 ng/mL，BPA、BPB、BPE、BPF、BPG的质量浓度为0.20~100 ng/mL，BPC的质量浓度为0.40~200 ng/mL，TBBPA的质量浓度为0.80~400 ng/mL，D_8_-BPS、D_5_-BPAP的质量浓度为2.0 ng/mL，^13^C_12_-BPA、D_8_-BPB、D_6_-BPZ的质量浓度为4.0 ng/mL，D_12_-BPE、D_10_-BPF、^13^C_12_-BPAF的质量浓度为10.0 ng/mL，D_6_-TBBPA的质量浓度为40.0 ng/mL。

### 1.3 样品前处理

采集市售30份预制菜样品，经过高速粉碎机混合均匀后，分装在干净的聚丙烯材质保鲜盒中，一分两份，一份检测，一份复检，于‒20 ℃冷冻保存，称样前，恢复到室温并混合均匀。

准确称取2.0 g（精确至0.001 g）混合均匀的样品，置于50 mL聚丙烯离心管中，加入200 μL混合标准同位素内标使用液，加入2.0 mL超纯水，涡旋振荡5 min使其完全分散，再加入8.0 mL乙腈，涡旋振荡20 min，再超声提取20 min后，以10 000 r/min离心10 min，取上清液过Captiva EMR-Lipid固相萃取柱，弃去前3.0 mL过滤液后，将剩余过滤液直接收集到进样瓶中供UPLC-MS/MS测定分析。

### 1.4 色谱条件

色谱柱：Waters ACQUITY HSS T_3_色谱柱（100 mm×2.1 mm，1.8 μm）；捕集柱：Waters ACQUITY BEH C_18_色谱柱（50 mm×2.1 mm，1.7 μm）；柱温：40 ℃；进样体积：2 µL；流动相A：0.01%氨水溶液，流动相B：甲醇；流速：0.2 mL/min。梯度洗脱条件：0~1.5 min，70%A~40%A；1.5~2.5 min，40%A；2.5~4.0 min，40%A~10%A；4.0~4.5 min，10%A~1%A；4.5~6.5 min，1%A；6.5~6.6 min，1%A~70%A；6.6~9.0 min，70%A。

### 1.5 质谱条件

电离源：ESI^-^，毛细管电压：2.5 kV，离子源温度：150 ℃，脱溶剂气温度：500 ℃，脱溶剂气流量：1 000 L/h，碰撞气流量：0.13 mL/min。15种双酚类化合物及9种同位素内标的定性、定量离子和碰撞能量见表1。

**表1:** 15种BPs及内标的质谱参数

Analyte	Retention time/min	Parent ion （*m/z*）	Product ions （*m/z*）	Declustering potential/V	Collision energies/eV	IS
Bisphenol A （BPA）	5.66	227.0	212.0^*^/133.0	46	18/26	^13^C_12_-BPA
Bisphenol B （BPB）	5.97	241.1	212.0^*^/226.0	44	18/18	D_8_-BPB
Bisphenol C （BPC）	6.18	255.1	240.1^*^/147.0	56	20/26	D_5_-BPAP
Bisphenol E （BPE）	5.36	213.1	198.1^*^/119.0	50	18/20	D_12_-BPE
Bisphenol F （BPF）	4.92	199.0	93.0^*^/105.0	46	22/22	D_10_-BPF
Bisphenol G （BPG）	6.65	311.2	296.1^*^/175.1	68	26/28	^13^C_12_-BPAF
Bisphenol M （BPM）	6.66	345.2	330.2^*^/251.5	70	30/30	D_5_-BPAP
Bisphenol P （BPP）	6.67	345.2	330.3^*^/133.0	68	26/38	^13^C_12_-BPAF
Bisphenol S （BPS）	2.67	249.0	108.0^*^/92.0	54	26/34	D_8_-BPS
Bisphenol Z （BPZ）	6.29	267.1	173.1^*^/145.1	62	28/36	D_6_-BPZ
Bisphenol AP （BPAP）	6.11	289.0	274.0^*^/195.0	48	22/28	D_5_-BPAP
Bisphenol AF （BPAF）	5.99	334.8	265.0^*^/197.0	40	26/40	^13^C_12_-BPAF
Bisphenol BP （BPBP）	6.43	351.1	273.8^*^/258.1	60	26/26	^13^C_12_-BPAF
Bisphenol FL （BPFL）	6.22	349.1	256.1^*^/215.1	62	28/28	D_5_-BPAP
Tetrabromobisphenol A （TBBPA）	3.95	542.8	419.8^*^/447.8	76	42/42	D_6_-TBBPA
^13^C_12_-Bisphenol A （^13^C_12_-BPA）	5.66	239.1	224.1^*^/139.0	50	18/28	-
D_8_-Bisphenol B （D_8_-BPB）	5.95	249.1	220.0^*^	44	18	-
D_12_-Bisphenol E （D_12_-BPE）	5.31	225.1	126.0^*^/97.0	50	26/26	-
D_10_-Bisphenol F （D_10_-BPF）	4.85	209.0	97.0^*^/110	54	22/18	-
D_8_-Bisphenol S （D_8_-BPS）	2.68	257.0	112.0^*^/160.0	54	26/22	-
D_6_-Bisphenol Z （D_6_-BPZ）	6.28	273.1	179.0^*^/225.0	62	24/32	-
D_5_-Bisphenol AP （D_5_-BPAP）	6.09	294.1	279.0^*^	48	22	-
^13^C_12_-Bisphenol AF （^13^C_12_-BPAF）	5.99	347.8	277.0^*^	40	26	-
D_6_-Tetrabromobisphenol A （D_6_-TBBPA）	3.95	548.8	448.5^*^/420.5	72	32/42	-

* Quantitative ion.

### 1.6 质量控制措施

为避免样品前处理所用器皿对实验本底的干扰，所有玻璃器皿参照文献［^30^］报道的方法进行清洗。移液器吸头、聚丙烯离心管在使用前用超纯水和甲醇各清洗3遍，Captiva EMR-Lipid固相萃取柱净化时，收集净化液之前，用3.0 mL提取液冲洗柱内壁。在实验过程中采用超纯水代替样品，作为过程空白样以监测本底。以同位素内标的回收率考察样品中目标物的萃取效率。上机测定时，每10份样品之间增加一个80%（v/v）乙腈水溶液空白样监测仪器背景。

## 2 结果与讨论

### 2.1 色谱柱的选择

比较了ACQUITY HSS T_3_色谱柱（100 mm×2.1 mm，1.8 μm）和ACQUITY BEH C_18_色谱柱（100 mm×2.1 mm，1.7 μm）对15种目标物的分离效果与响应值，发现上述两款色谱柱均可以分离15种BPs，甲醇-氨水溶液为流动相时，BPS在BEH C_18_柱上保留较弱，出峰时间比较靠前，保留时间仅为0.9 min，易受到溶剂效应的影响，影响定量的准确性，改用HSS T_3_色谱柱时，BPS的保留时间为2.67 min，且所有BPs目标物在HSS T_3_上的响应值高于BEH C_18_柱，故确定HSS T_3_色谱柱（100 mm×2.1 mm，1.8 μm）为最佳分离色谱柱。

### 2.2 实验本底的控制

液相系统管路等部件中痕量BPA本底对实验产生干扰，影响方法的准确定量，实验发现，在自动进样器的前端连接一根BEH C_18_柱（50 mm×2.1 mm，1.7 μm）作为捕集柱，可将部件中痕量的BPA 捕获在此柱上，经流动相洗脱，利用时间差，将样品中目标物BPA与仪器系统中痕量的BPA分离。以80%（v/v）乙腈水溶液为实验对象考察仪器中BPA的本底，如图1a所示，未加捕集柱时，管路中痕量BPA与样品中目标物未完全分离，影响BPA结果的准确性，增加捕集柱后，该现象得到明显改善，如图1b所示，管路中痕量BPA与目标物完全分离，解决了系统管路带来的BPA干扰问题。

**图1:**
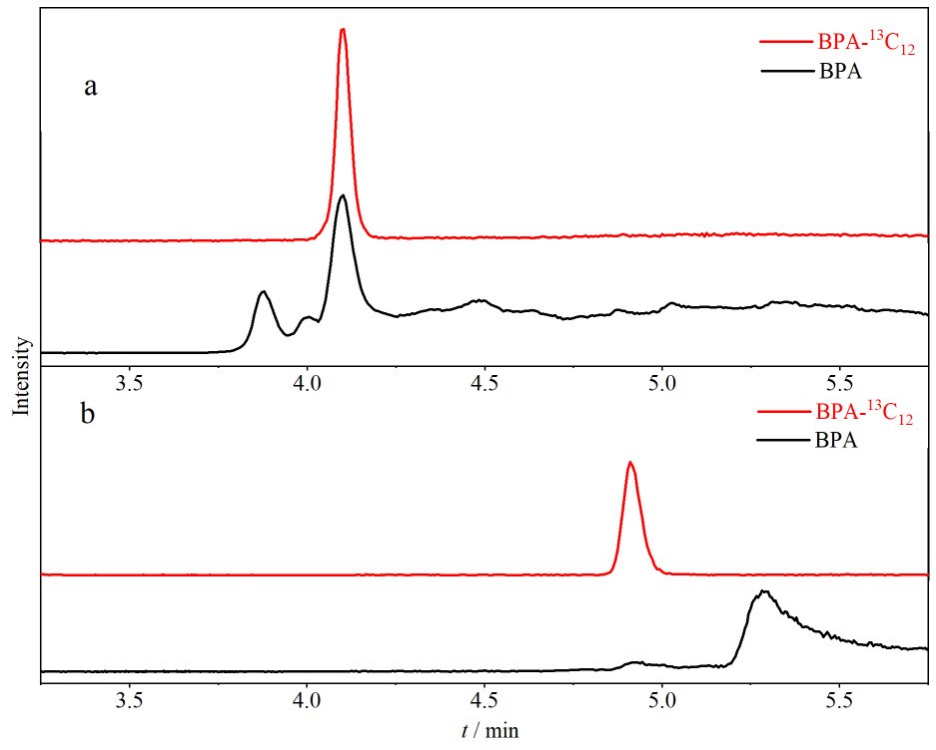
连接捕集柱（a）前、（b）后仪器管路中BPA的色谱图

实验过程中，按照1.6节进行质量控制，以超纯水代替样品，按照1.3节方法进行前处理，上机测定，过程空白的相关谱图见附图S1（https：//www.chrom-China.com），目标物的检测结果均小于该方法的检出限，过程中带入的本底均不影响方法的定量。

### 2.3 流动相的优化

考察了甲醇-水、乙腈-水对15种BPs响应值及分离度的影响，结果表明，甲醇-水为流动相时，各目标物的响应值明显优于乙腈-水。负离子模式下，流动相水相中加入适量氨水有利于目标物的电离，可提高目标物的响应值。以甲醇为有机相，考察了不同体积分数（0.001%、0.005%、0.01%、0.05%和0.1%）的氨水溶液对15种BPs响应值及峰形的影响。结果表明，当氨水体积分数较低时（小于0.01%），BPS的响应值更高，且在HSS T_3_色谱柱上有更好的保留，保留时间为2.67 min，但此条件下，TBBPA出现峰形展宽。随着氨水体积分数的增加，TBBPA峰形有所改善，当氨水体积分数大于0.01%时，TBBPA峰形对称、尖锐，但BPS的响应值呈现下降趋势，综合考虑，选择甲醇-0.01%氨水溶液作为最佳流动相。

### 2.4 样品提取液的选择

动物性食品富含蛋白质，采用纯有机溶剂作为提取液时，沉淀蛋白质速度快易使样品结团，目标物被包裹在样品中，不能被提取液提取出来，影响提取效率。本研究选取猪肚鸡、红烧肉、肉包子和梅菜扣肉为基质样品，加入2.0 mL超纯水将样品分散后，再加入8.0 mL有机溶剂。比较了乙腈、甲醇、甲基叔丁基醚^［31］^、乙酸乙酯^［32］^等溶剂对15种BPs的提取效果。因甲基叔丁基醚和乙酸乙酯需要经过氮吹复溶才能进入质谱测定，氮吹过程会造成目标物的损失和时间成本的增加，且有较大的挥发性气味，不适于样品的批量处理。与甲醇相比，乙腈作为提取液，具有很好的沉淀蛋白质的优势，提取效果更好，故选用乙腈作为提取液。加入2.0 mL超纯水分散样品，考察加入不同体积的乙腈（2.0、4.0、6.0、8.0 mL）对目标物提取效果的影响，结果如图2所示，加入乙腈体积为8.0 mL时，4种基质中各目标物的峰面积大多达到最大值，选择8.0 mL乙腈作为最优提取溶剂。

**图2:**
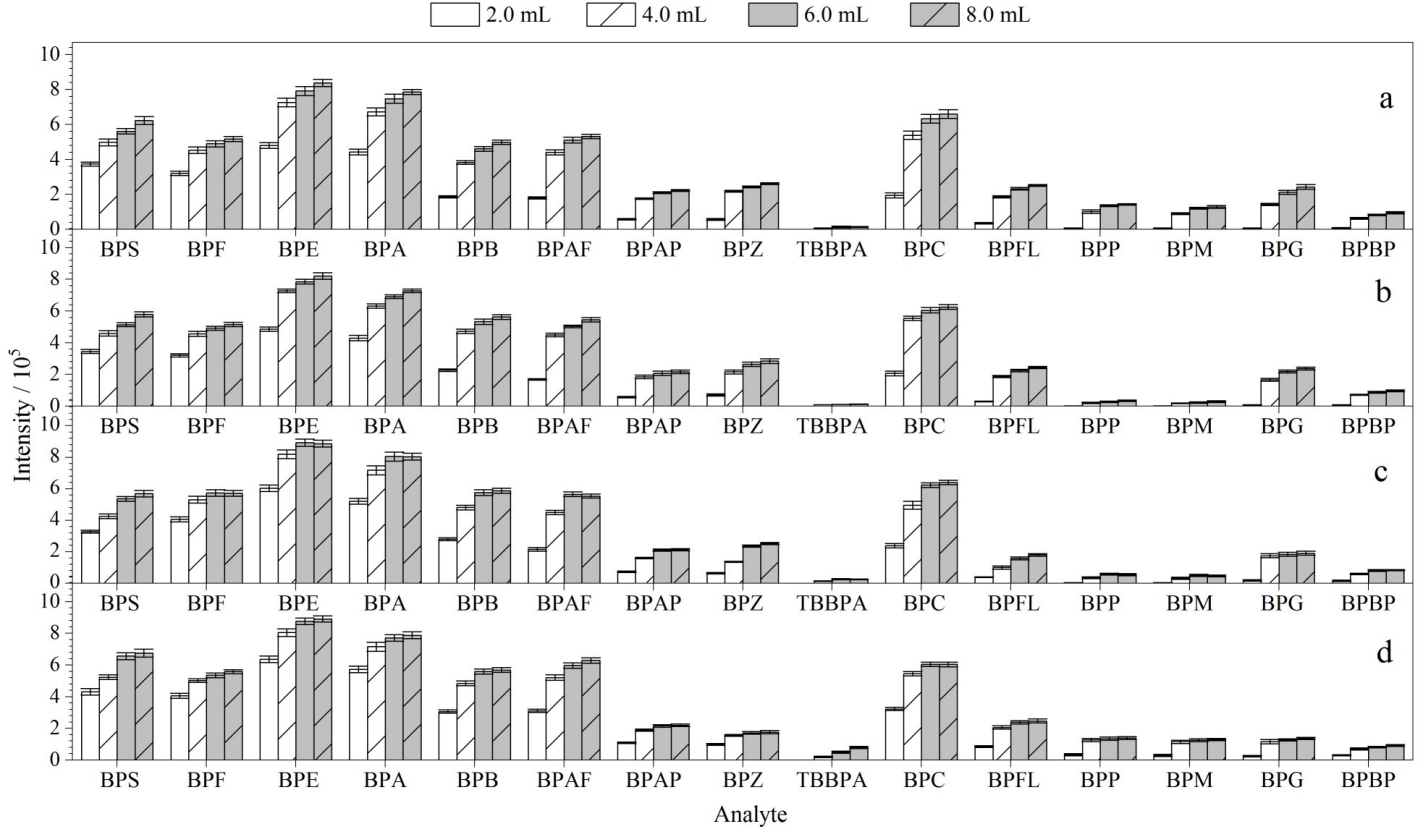
不同体积乙腈对15种BPs响应值的影响（*n*=3） a. pork belly chicken matrix； b. braised pork matrix； c. meat bun matrix； d. pork with salted vegetable matrix.

### 2.5 净化方法的优化

QuEChERS 法以快速、简单、有效、可靠和安全为优势，被广泛用于复杂基质的前处理，Captiva EMR-Lipid柱、PRiME HLB柱净化被称为“一步法”净化，除有效除去干扰物外，操作步骤简单，适用于批量样本的同时测定。本研究比较了QuEChERS法和Captiva EMR-Lipid柱、PRiME HLB柱净化对15种BPs净化效果的影响（见图3）。具体的优化步骤如下：QuEChERS法参考文献［^11^］报道的方法，向预制菜样品（梅菜扣肉）中添加10 µg/kg的混合标准溶液，再按照1.3节加入混合标准同位素内标使用液，加入2.0 mL超纯水涡旋分散后，再加入8.0 mL乙腈，涡旋振荡、超声提取，分别取1.0 mL离心后的提取液加入装有50 mg PSA、50 mg C_18_的净化管中净化，结果发现PSA不仅无法很好地除去样品中的色素，还影响TBBPA的回收率，可能因为TBBPA中的溴原子和甲基具有电子接受能力，而PSA中的氨基可以作为电子供体，二者之间可以通过电子供体-受体相互作用实现吸附，说明PSA不适合TBBPA的净化。采用C_18_吸附剂净化时，TBBPA、BPM、BPG的回收率低于80%，使用PRiME HLB柱净化时，BPFL、BPP、BPG、BPBP的加标回收率均低于50%，BPC回收率高于120%，Captiva EMR-Lipid固相萃取柱净化后的15种BPs回收率范围为80%~120%，因此最终选择Captiva EMR-Lipid固相萃取柱作为最佳净化方法。

**图3:**
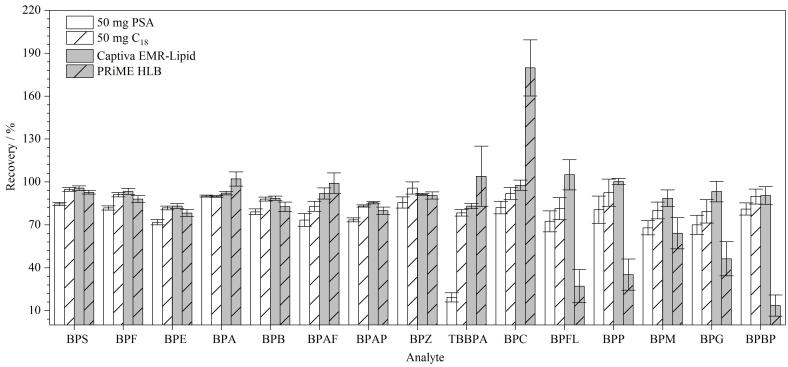
采用不同净化方式时15种BPs的回收率（*n*=3）

### 2.6 基质效应（ME）

因食品样品成分较为复杂，样品基质很有可能对目标物离子产生抑制或者增强效应。本研究中，ME定义为（基质配制校准曲线斜率-溶剂标准曲线斜率）/溶剂标准曲线斜率×100%。当ME=0时，无基质效应；当ME<0时，为基质抑制；当ME>0时，为基质增强；当|ME|≤20%时，可认为无明显的基质效应影响^［33］^。随着样品质量的增加，15种BPs会产生不同程度的基质增强或抑制效应。故本实验优化了实际样品的取样量，分别称取1.0、2.0、5.0 g预制菜样品，采用上述1.3节方法处理，获得空白基质提取液，用该基质液配制校准溶液，计算ME值，如图4所示。ME值大小随着称样量的变化而变化，如BPB的基质效应随着称样量的增加，由基质增强逐渐变为基质抑制，这可能与基质的浓度大小有关系。称样量为5.0 g时，BPB、BPAF、BPC、BPFL和BPM的ME值大于20%，有较强的基质效应；考虑到取样量较少时，样品不具有代表性；当称样量为2.0 g时，|ME|均小于20%，无明显的基质效应，最终选择样品称样量为2.0 g，加入同位素内标，溶剂标准曲线进行定量。

**图4:**
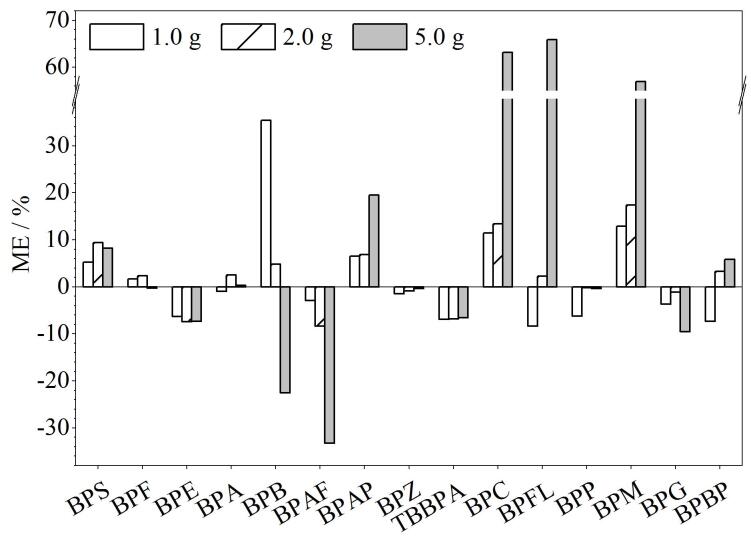
不同样品质量下15种BPs的基质效应

### 2.7 方法性能

#### 2.7.1 线性范围、检出限与定量限

按照1.2节配制混合标准系列，上机测定，分别以目标物的质量浓度为横坐标（*x*，ng/mL），以目标物的峰面积与同位素内标峰面积比值为纵坐标（*y*），绘制标准曲线。结果表明，15种BPs在各自范围内具有良好的线性关系，相关系数（*R* ^2^）均大于0.999 0。分别以3倍和10倍信噪比（*S/N*）确定方法的检出限（LOD）和定量限（LOQ），15种BPs的LOD为0.01～0.45 μg/kg，LOQ为0.03～1.50 μg/kg，如表2所示。

**表2:** 15种BPs的线性范围、线性方程、相关系数、检出限和定量限

Analyte	Linear range/（ng/mL）	Linear equation	*R* ^2^	LOD/（μg/kg）	LOQ/（μg/kg）
BPA	0.20-100	*y*= 0.2776*x*+0.06147	0.9996	0.25	0.80
BPB	0.20-100	*y*= 0.3097*x*+0.00793	0.9999	0.45	1.50
BPC	0.40-200	*y*= 0.1230*x*+0.00859	0.9999	0.03	0.10
BPE	0.20-100	*y*= 1.3523*x*+0.08495	0.9996	0.30	1.00
BPF	0.20-100	*y*= 0.1468*x*+0.01209	0.9999	0.45	1.50
BPG	0.20-100	*y*= 0.0886*x*+0.00644	0.9991	0.08	0.25
BPM	0.02-10.0	*y*= 0.8630*x*‒0.00244	0.9993	0.25	0.80
BPP	0.02-10.0	*y*= 0.6360*x*+0.00209	0.9998	0.10	0.30
BPS	0.02-10.0	*y*= 0.9807*x*+0.21726	0.9998	0.03	0.10
BPZ	0.04-20.0	*y*= 0.2759*x*+0.00192	0.9999	0.06	0.20
BPAP	0.04-20.0	*y*= 0.4214*x*+0.00091	0.9996	0.01	0.03
BPAF	0.02-10.0	*y*= 1.3539*x*+0.01892	0.9992	0.08	0.25
BPBP	0.04-20.0	*y*= 0.1868*x*+0.00107	0.9991	0.07	0.22
BPFL	0.04-20.0	*y*= 0.5773*x*+0.00461	0.9998	0.04	0.14
TBBPA	0.80-400	*y*= 0.0337*x*‒0.00213	0.9991	0.40	1.30

*y*： ratio of peak area of analyte to internal standard； *x*： mass concentration， ng/mL.

#### 2.7.2 回收率和精密度

按1.3节描述的前处理方法，选择阴性预制菜样品为基质，进行3个水平的加标回收试验，每个加标水平做6次平行测试。结果表明，在低、中、高3个加标水平下，15种BPs的加标回收率为70.9%～105.8%，精密度为0.6%～9.1%（见表3）。本方法适用于预制菜食品中15种BPs的检测分析。

**表3:** BPs在3个水平下的加标回收率和精密度 (*n*=6)

Analyte	Background/(μg/kg)	Added/(μg/kg)	Found/(μg/kg)	Recovery/%	RSD/%	Analyte	Background/(μg/kg)	Added/(μg/kg)	Found/(μg/kg)	Recovery/%	RSD/%
BPA	<0.25	2.5	1.973	78.9	3.8	BPS	<0.03	0.25	0.222	88.8	5.6
		25	22.720	90.9	2.4			2.5	2.127	85.1	3.2
		250	233.979	93.6	1.4			25	25.026	100.1	1.4
BPB	<0.45	2.5	2.183	87.3	5.3	BPZ	<0.06	0.5	0.477	95.4	6.2
		25	22.958	91.8	1.7			5.0	4.801	96.0	1.2
		250	226.270	90.5	3.3			50	48.200	96.4	0.6
BPC	<0.03	5.0	3.996	79.9	3.5	BPAP	<0.01	0.5	0.418	83.5	5.9
		50	50.111	100.2	2.4			5.0	5.087	101.8	2.7
		500	529.002	105.8	1.7			50	48.423	96.8	1.1
BPE	<0.30	2.5	2.120	84.8	2.3	BPAF	<0.08	0.25	0.194	77.7	2.1
		25	22.833	91.3	2.2			2.5	2.222	88.9	1.1
		250	235.466	94.2	2.3			25	22.888	91.6	1.4
BPF	<0.45	2.5	2.224	89.0	3.6	BPBP	<0.07	0.5	0.429	85.9	9.1
		25	23.942	95.8	1.1			5.0	4.805	96.1	2.0
		250	241.261	96.5	1.4			50	44.405	88.8	3.4
BPG	<0.08	2.5	2.203	88.1	3.1	BPFL	<0.04	0.5	0.390	77.9	2.6
		25	23.508	94.0	4.3			5.0	3.547	70.9	2.6
		250	217.095	86.8	4.1			50	37.857	75.7	1.4
BPM	<0.25	0.25	0.245	98.1	4.8	TBBPA	<0.40	10	8.979	89.8	3.4
		2.5	2.471	98.8	1.7			100	84.188	84.1	1.2
		25	24.338	97.4	3.0			1000	872.340	87.2	1.5
BPP	<0.10	0.25	0.194	77.7	5.0						
		2.5	2.035	81.4	2.3						
		25	18.834	75.3	4.2						

#### 2.7.3 方法性能比较

将建立的检测方法与文献报道和标准检测方法比较分析，如表4所示。本方法中BPA的检出限低于李星等^［5］^、周勇等^［32］^、周健等^［34］^报道的方法；BPB、BPC、BPAF的检出限低于王斌等^［10］^报道的方法；BPAF、BPAP的检出限低于Tan等^［35］^报道的方法；与Tan等^［35］^报道的方法相比，本方法的BPA、BPB、BPF、BPP、BPS、BPZ的检出限略高，这可能与样品基质有一定关系。此外，本方法中涵盖BPs的种类多于报道方法。现行有效的标准检测方法中，多数是食品接触材料中双酚类化合物迁移量的测定及水质中双酚类化合物的检测，无预制菜基质的标准检测方法。GB 31660.2-2019^［36］^仅规定了水产品中BPA的测定方法，该标准采用乙酸乙酯提取水产品，凝胶渗透色谱和固相萃取净化，七氟丁酸酐衍生，气相色谱-质谱法测定，外标法定量，样品前处理耗时，环境污染大，不适合批量样品的测定。本研究充分利用Captiva EMR-Lipid柱净化法的优点，有效除去了样品中的油脂，适用于预制菜样品的前处理，样品提取液净化后直接进样，简化了前处理步骤，同位素内标的使用，有效降低了基质效应，提高了方法的准确性。

**表4:** 不同基质样品中BPs分析方法的比较

Matrices	Analytes	Extraction and purification	Instrumental method	Recovery （RSD）/%	LOD/（μg/kg）	Ref.
Milk	BPA， BPF， BPS	liquid-liquid extraction-PRiME HLB solid phase extraction	LC-MS/MS	90.4-103.1（2.76-7.34）	1.0	［^5^］
Fruits and vegetables	BPA， BPB， BPF， BPP， BPS， BPZ， BPAF， BPAP	liquid-liquid extraction-QuEChERS	UPLC-MS/MS	66-118 （2-20）	0.01-0.10	［^8^］
Egg Yolk filling	BPA， BPB， BPC， BPF， BPS， BPAF	C_18_ dispersion adsorbent solid phase extraction	LC-MS/MS	82.1-113.2 （2.70-9.50）	0.50-5.0	［^10^］
Canned tuna	BPA	liquid-liquid extraction	UHPLC-MS/MS	80.4-88.9 （5.12-6.08）	0.2	［^32^］
Canned meats	BPA， BPS	liquid-liquid extraction-QuEChERS	LC-MS/MS	95.1-108 （2.0-5.4）	0.10-0.50	［^34^］
Meats	BPA， BPB， BPF， BPP， BPS， BPZ， BPAF， BPAP	liquid-liquid extraction-QuEChERS	UPLC-MS/MS	70.1-115.5 （1.1-10.3）	0.01-0.11	［^35^］
Aquatic products	BPA	gel purification and HLB solid phase extraction	GC-MS/MS	70.0-110	0.10	［^36^］
Prepared dishes	BPA， BPB， BPC， BPE， BPF， BPG， BPM， BPP， BPS， BPZ， BPAP， BPAF， BPBP， BPFL， TBBPA	liquid-liquid extraction-Captiva EMR-lipid purification	UPLC-MS/MS	70.9-105.8 （0.6-9.1）	0.01-0.45	this study

### 2.8 实际样品的测定

采用建立的检测方法分析了市售的30份预制菜食品（S1~S30）中的15种BPs，包括猪肚鸡、红烧肉、肉包子、梅菜扣肉、佛跳墙和毛血旺等基质，16.7%的样品中检出了2种BPs，实际样品S7中同时检出BPA与BPS，色谱图见图5。11份样品分别检出BPA、BPB、BPC、BPG、BPS和BPAF，其中BPA的检出率最高，为16.7%，其次BPC、BPG、BPB、BPAF和BPS的检出率分别为10.0%、10.0%、6.67%、6.67%和3.33%，检测结果如表5所示。样品中BPG、BPB、BPA、BPC、BPS和BPAF含量的中位值分别为4.15、3.25、2.68、2.16、1.49和0.47 μg/kg。

**图5:**
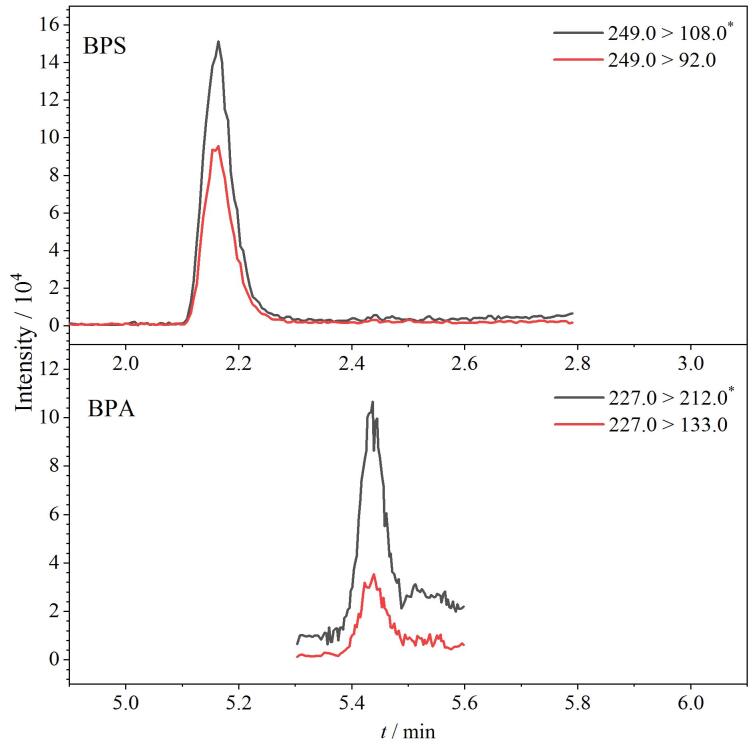
实际样品（S7）中BPs的色谱图

**表5:** 实际样品中15种BPs的测定结果

Analyte	Contents/（μg/kg）											Detection rate/%	Average value/（μg/kg）
	S1	S2	S3	S4	S5	S6	S7	S8	S9	S10	S11		
BPA	-	-	10.8	-	-	1.30	0.617	-	0.380	-	0.305	16.7	2.68
BPB	-	-	-	-	3.89	-	-	-	-	2.62	-	6.67	3.25
BPC	2.65	3.17	-	-	-	-	-	-	-	-	0.645	10.0	2.16
BPE	-	-	-	-	-	-	-	-	-	-	-	-	-
BPF	-	-	-	-	-	-	-	-	-	-	-	-	-
BPG	-	-	9.25	3.06	-	-	-	0.138	-	-	-	10.0	4.15
BPM	-	-	-	-	-	-	-	-	-	-	-	-	-
BPP	-	-	-	-	-	-	-	-	-	-	-	-	-
BPS	-	-	-	-	-	-	1.49	-	-	-	-	3.33	1.49
BPZ	-	-	-	-	-	-	-	-	-	-	-	-	-
BPAP	-	-	-	-	-	-	-	-	-	-	-	-	-
BPAF	-	-	-	-	0.280	-	-	-	-	0.659	-	6.67	0.470
BPBP	-	-	-	-	-	-	-	-	-	-	-	-	-
BPFL	-	-	-	-	-	-	-	-	-	-	-	-	-
TBBPA	-	-	-	-	-	-	-	-	-	-	-	-	-

-： <LOD.

## 3 结论

本研究通过对色谱、质谱参数和固相萃取条件进行了优化，建立了超高效液相色谱-串联质谱法检测预制菜食品中15种BPs残留量的分析方法。该方法采用Captiva EMR-Lipid固相萃取柱净化提取液，有效解决了预制菜食品中高油脂干扰的问题，充分发挥了Captiva EMR-Lipid柱的高效脱脂优势，提取效率高，同时，稳定同位素内标的使用，有效降低了复杂食品的基质效应，检测结果定量准确。将所建立的方法对实际样品进行测定，发现BPs在预制菜食品中有残留。此前欧盟在2024年2月9日出台新的草案，该倡议禁止在食品接触材料（FCM）中使用 BPA，包括塑料和涂层包装以及限制其他双酚及其衍生物的使用^［37］^。本文建立的分析方法测定的目标物多，前处理方法简单，灵敏度高，定量准确，适用于预制菜食品样品中15种BPs的定性筛查和定量分析，为研究其在预制菜食品中的残留和风险评估提供了可靠的技术支持。
